# Sleep and mental health in childhood: a multi-method study in the general pediatric population

**DOI:** 10.1186/s13034-022-00447-0

**Published:** 2022-02-17

**Authors:** Elisabet Blok, M. Elisabeth Koopman-Verhoeff, Daniel P. Dickstein, Jared Saletin, Annemarie I. Luik, Jolien Rijlaarsdam, Manon Hillegers, Desana Kocevska, Tonya White, Henning Tiemeier

**Affiliations:** 1 Department of Child and Adolescent Psychiatry/Psychology, Erasmus MC-Sophia Children’s Hospital, University Medical Center, Rotterdam, The Netherlands; 2 The Generation R Study Group, Erasmus MC, University Medical Center, Rotterdam, The Netherlands; 3 Institute of Education and Child Studies, Leiden University, Leiden, The Netherlands; 4 Emma Pendleton Bradley Hospital, East Providence, RI USA; 5 PediMIND Program, McLean Hospital, Harvard Medical School, Boston, MA USA; 6 Simches Center of Excellence in Child and Adolescent Psychiatry, McLean Hospital, Harvard Medical School, Boston, MA USA; 7 Department of Psychiatry and Human Behavior, Alpert Medical School of Brown University, Providence, RI USA; 8 Department of Epidemiology, Erasmus MC, University Medical Center, Rotterdam, The Netherlands; 9 Department of Sleep and Cognition, Netherlands Institute for Neuroscience, An Institute of the Royal Netherlands Society for Arts and Sciences, Amsterdam, The Netherlands; 10 Department of Radiology and Nuclear Medicine, Erasmus MC, University Medical Center, Rotterdam, The Netherlands; 11 Department of Social and Behavioral Science, Harvard T.H. Chan School of Public Health, Boston, MA USA

**Keywords:** Psychopathology, Insomnia, Actigraphy, Development, Sleep, Mood

## Abstract

**Background:**

Sleep problems, altered sleep patterns and mental health difficulties often co-occur in the pediatric population. Different assessment methods for sleep exist, however, many studies only use one measure of sleep or focus on one specific mental health problem. In this population-based study, we assessed different aspects of sleep and mother-reported mental health to provide a broad overview of the associations between reported and actigraphic sleep characteristics and mental health.

**Methods:**

This cross-sectional study included 788 children 10-11-year-old children (52.5% girls) and 344 13–14-year-old children (55.2% girls). Mothers and children reported on the sleep of the child and wrist actigraphy was used to assess the child’s sleep patterns and 24 h activity rhythm. Mental health was assessed via mother-report and covered internalizing, externalizing and a combined phenotype of internalizing and externalizing symptoms, the dysregulation profile.

**Results:**

Higher reported sleep problems were related to more symptoms of mental health problems in 10–11- and 13–14-year-old adolescents, with standardized ß-estimates ranging between 0.11 and 0.35. There was no association between actigraphy-estimated sleep and most mental health problems, but earlier sleep onset was associated with more internalizing problems (ß = − 0.09, SE = 0.03, p-value = 0.002), and higher intra-daily variability of the 24 h activity rhythm was associated with more dysregulation profile symptoms at age 10–11 (ß = 0.11, SE = 0.04, p-value = 0.002).

**Discussion:**

Reported sleep problems across informants were related to all domains of mental health problems, providing evidence that sleep can be an important topic to discuss for clinicians seeing children with mental health problems. Actigraphy-estimated sleep characteristics were not associated with most mental health problems. The discrepancy between reported and actigraphic sleep measures strengthens the idea that these two measures tap into distinct constructs of sleep.

**Supplementary Information:**

The online version contains supplementary material available at 10.1186/s13034-022-00447-0.

## Background

Sleep problems and mental health difficulties often co-occur in childhood and adolescence [1, 2]. Children transitioning from late childhood into early adolescence, undergo rapid biological and emotional changes [3, 4], including changes in sleep [5]. Thus, studying sleep in relation to mental health in preadolescence and early adolescence has the potential to provide insight into how sleep and behavior are related. Understanding how sleep is related to mental health is of clinical relevance. First, sleep problems can be an early and sensitive marker for parents and clinicians to help identify emerging mental health problems. Second, sleep is a modifiable factor [6], making it a potential target for different treatment strategies.

The relationship between sleep and psychopathology has been studied extensively in adults. Sleep problems are a common complaint across psychiatric diagnoses [1, 7], including disorders in the internalizing domain (e.g., depression [8–11] and anxiety [11–13]), the externalizing domain (e.g., ADHD [14, 15] and aggression [16]), and disruptive mood dysregulation disorder (DMDD) [17, 18], a diagnosis with characteristics of both the internalizing and externalizing domains. However, it is important to note that the relationship between sleep and mental health is not consistent across sleep measurements used. Studies focusing on reported sleep problems, measured via questionnaire completed by the child or the parent, have consistently demonstrated more sleep problems in children with mental health problems across domains of psychopathology [8, 9, 11–17]. In contrast, results of studies based on objective sleep measures, such as polysomnography and actigraphy, are less consistent. Using actigraphy, both sleep patterns and 24 h activity rhythm measures can be obtained. Regarding sleep patterns, some studies report no associations between depression, ADHD and DMDD and objective sleep measures [19–21]. Yet, other studies indicate reduced total sleep time in children with ADHD [14] and increased total sleep time in adolescents with depression [10]. Additionally, reduced sleep efficiency is observed in children with ADHD [14], depression [9] and DMDD [18]. The relationship between the 24 h activity rhythm and mental health has not been widely studied, even though significant changes in the 24 h activity rhythm occur in early adolescence [5]. For example, the 24 h activity rhythm shifts to a later phase, which in combination with increased autonomy could lead to a lower interdaily stability (i.e., less regularity) of the 24 h activity rhythm. Indeed, a lower interdaily stability has been observed in adolescents as compared to children [22].

Despite previous studies on sleep and mental health in children, several knowledge gaps remain. First, results have been shown to vary depending on the sleep measure collected and reporter [23–26], but we are not aware of studies which have assessed both self- and mother-reported sleep problems as well as actigraphic sleep measures together with mental health within one sample. Comparing results using multiple assessment methods and raters within the same sample can elucidate whether prior inconsistent findings can be explained by the choice of assessment or reporter. Second, the current literature lacks studies on the associations between the 24 h activity rhythm and mental health problems. Third, earlier work provided evidence that the association between sleep and depression appeared stronger in adolescents than in children [27]. Potentially, this is also true for other mental health problems in late childhood and early adolescence. Lastly, the relationship between mental health problems and sleep might be specific to certain mental health difficulties (e.g. depressive symptoms are related to longer sleep duration whereas ADHD symptoms are related to a shorter sleep duration [10, 14]). Unraveling whether the relationship between mental health and different sleep aspects is similar across or specific to mental health domains can improve our understanding of co-occurrence and has the potential to ultimately guide the clinical practice. Thus, in this study we incorporate both self- and mother-reported perceived sleep problems, and actigraphic measures of sleep patterns and the 24 h activity rhythm to study how mental health problems relate to these measures across late childhood and early adolescence. We focus on continuous measures of three broad domains of mental health, including an internalizing domain, an externalizing domain and a domain that has internalizing and externalizing features called the dysregulation profile [28].

The primary aim of this study is to assess the relationship between sleep and mental health, incorporating a wide range of sleep measures, including both reported as well as actigrapic sleep measures, and broad domains of mental health across late childhood and early adolescence in a large population based cohort. Our primary analyses included both confirmatory and exploratory analyses. Regarding confirmatory analyses, we hypothesized that all domains of mental health would be associated with reported sleep problems [8, 9, 11–17]. For the actigraphy measures we hypothesized that longer total sleep time would be associated with internalizing symptoms [10] and shorter sleep duration with externalizing symptoms and the dysregulation profile [14]. Further we expected that all domains of mental health would be associated with reduced sleep efficiency [9, 14, 18]. Since no prior work has focused on the relationship between mental health and the 24 h activity rhythm in children, we include these as exploratory analyses. As a secondary aim of this study, we employ exploratory analyses to assess mental health domains that are not covered by the broad internalizing, externalizing and dysregulation profile domains, assessing the relationships between sleep and thought problems and social problems.

## Methods

### Participants

**Fig. 1:**
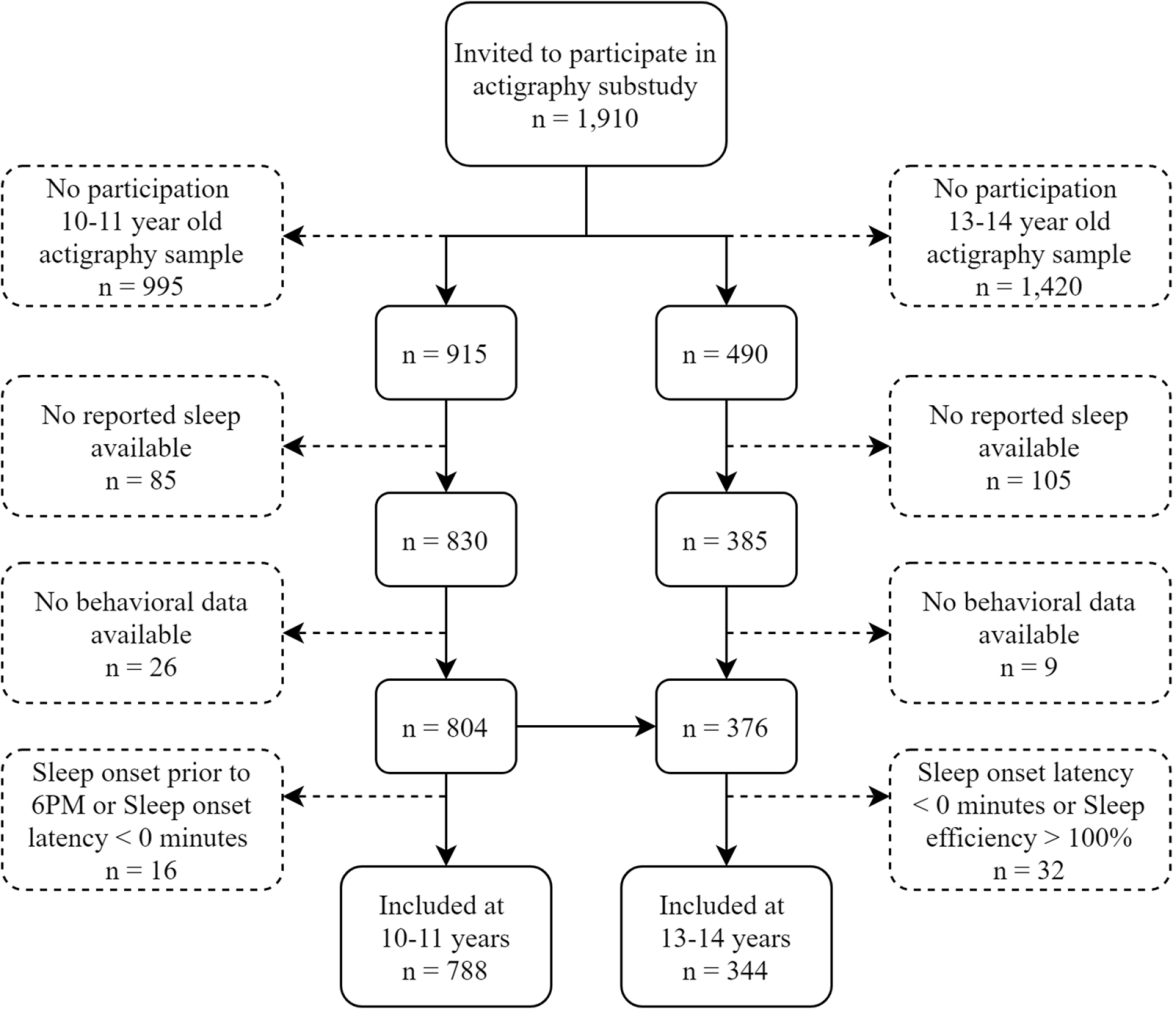
Flowchart of the study sample

Participants were drawn from the Generation R Study, a birth cohort from Rotterdam, the Netherlands. All pregnant women with a delivery date between April 2002 and January 2006 were invited to participate [29]. Since recruitment, the children and their families have been invited for multiple waves of data collection. At 10 years of age 8548 children were invited to visit the research center, of those, 7968 were invited again at 13 years of age. Actigraphic sleep patterns were estimated in a subsample of Generation R, for which in total 1910 children were invited in two separate waves. A detailed description of the sampling strategy for this subsample has been described previously [30]. Briefly, included participants had high follow-up rates, premature born participants were oversampled, and were more often of Western decent. The first actigraphy data collection started nearly 1 year after the 10-year assessment (n = 915) and the second was conducted nearly 1 year after the 13-year assessment (n = 490), these assessments included no repeated measurements. Children were excluded from analyses if the reported sleep or behavioral data was missing at the corresponding age and when outliers corresponding to impossible or highly unlikely values were detected, resulting in a total of 788 children who were included at 10–11 years of age and 344 children at 13–14 years of age. Figure 1 presents a flowchart of the study sample. The Medical Ethics Committee of the Erasmus Medical Center approved all study procedures, and all parents and participants provided written informed consent or assent if appropriate.

### Instruments

#### Child behavior checklist (CBCL)

The CBCL is a widely used parent-reported screening questionnaire to assess child behavior. The CBCL version 6–18 [31] was used in both waves and consists of 112 items scored on a three point Likert scale (0 = not true, 1 = somewhat true, 2 = very/often true). It is a reliable and valid questionnaire assessing mental health problems in school age children and adolescents [31]. The questionnaire was completed at both age waves by the primary caregiver, which was in the majority of the cases the mother. Specifically, at 10 years of age all data was obtained from maternal report, and at 13 years of age, 8.3% of the CBCL reports were obtained from fathers. Symptom severity is scored with eight empirically derived syndrome scales, being anxious/depressed, withdrawn/depressed, somatic complaints, social problems, thought problems, attention problems, rule-breaking behavior, and aggressive behavior. From these syndrome scales, three broadband scales can be derived, the internalizing scale consists of anxious/depressed, withdrawn/depressed and somatic complaints, (range 0–64), the externalizing scale consists of rule-breaking behavior, and aggressive behavior (range 0–70) and the dysregulation profile scale comprises subscales from both domains as it is a sum score of the anxiety/depressed, attention problems, and aggressive behavior scales (range 0–82), with a higher score reflecting more problems. In the current sample, the dysregulation profile is highly correlated with the total problems score of the CBCL, that includes all 113 items, at both age waves the correlation is 0.96. Further, since this study is embedded in a population-based study, all measures of mental-health were positively skewed. Given that the range was different for the broad domains of mental health, we standardized all mental health measures to a mean of 0 with a standard deviation of 1.

#### Actigraphy-estimated sleep

Sleep patterns and the 24 h activity rhythm were estimated with wrist tri-axial actigraphy (GENEActiv; Activinsights, UK) worn on non-dominant wrist for 9 subsequent days (5 school days and 4 weekend days) [32]. Each morning children filled out sleep diaries answering questions about their sleep timing (e.g., the time they went to bed). All data from the diaries was manually entered in a database and checked for outliers; no human censoring of bedtime/rise-time was performed. This data was used as input to guide fully automated actigraphy analyses. The binary files were processed using the R-package GGIR [33]. This procedure generated the following sleep measures: total sleep time, sleep onset, sleep onset latency, sleep efficiency, and the total number of awakenings after sleep onset. Total sleep time is the duration of epochs classified as sleep during the night. Sleep onset is the time of falling asleep. Sleep onset latency indicates the time between the reported bedtime and the sleep onset. Sleep efficiency is the total sleep time divided by time in bed. Additionally, we calculated 24 h activity rhythm parameters: the interdaily stability (IS), the intradaily variability (IV), and the onset of the least active 5-h period in 24-h (L5 onset), according to a method described by Van Someren et al. [34]. Due to the nonparametric nature of the variables no specific waveform is assumed. IS indicates the resemblance of the rhythm between different days. IV indicates the fragmentation of the rhythm to its 24 h amplitude; i.e., the frequency of alterations between active and rest states. L5 is defined as resting levels during the 5 h period when there was the least activity, the L5 onset indicates the timing of the 24 h activity rhythm. Most actigraphy-estimated sleep measures were normally distributed, with the exception of the sleep onset latency, which was right-skewed.

#### Self reported sleep problems (10–11 years)

Child reported sleep problems were assessed by self-report questionnaire with six questions about their perceived sleep in general: “Do you find it difficult to go to bed?”; “Do you find it difficult to fall asleep?”; “Do you think you get enough sleep?”; “If you wake up at night, do you find it difficult to fall asleep again?”; “Do you feel rested when you wake in the morning?”; “When you come out of your bed in the morning, do you feel rested?”. These questions were derived from the widely used Sleep Disturbance Scale for Children (SDSC) [35] and slightly rephrased for our pediatric population. Similar questions can be found in other sleep scales for children such as the Sleep Self Report [36], and the Sleep Habits Survey [37]. The three possible responses for each item: “No”, “Sometimes” or “Yes” were scored on a Likert scale. Responses from all six items were summed to calculate a total score; the total score had an internal consistency of α = 0.65; higher scores indicate more sleep problems.

#### Self reported sleep problems (13–14 years)

Child reported sleep problems in general were assessed via a questionnaire consisting of questions derived from the Sleep Habits Survey (SHS) [37] and the Youth Self Report (YSR) [31]. Sleep problems were assessed using four items from the SHS: “How many minutes do you need to fall asleep after the light is shut off”; “How many times do you wake up during the night?” (never, once, two or three times, more than three times); “Do you feel like you normally sleep enough?” (too little, enough, too much); “Do you think you are a good or a poor sleeper?”, and two items from the YSR: “I sleep less than most boys and girls” and “I have trouble sleeping”. Items were individually standardized between 0 and 1, to give all items the same the same weight and summed to make a total sleep problems score; the total score had an internal consistency of α = 0.69; higher scores indicate more sleep problems. For ease of comparison, we standardized both sleep problem scale scores.

#### Mother-reported sleep problems

Mother-reported sleep problems were obtained during a structured interview that took place during the home-visits performed in the actigraphy subsample. Mothers were asked to report their children’s experienced difficulties initiating or maintaining sleep by using this subscale of the Sleep Disturbance Scale for Children (SDSC) [35]. Seven items were included: “How many hours of sleep does your child get on most nights”, “How long after going to bed does your child usually fall asleep”, “The child goes to bed reluctantly”, “The child has difficulty getting to sleep at night”; “The child feels anxious or afraid when falling asleep”, “The child wakes up more than twice per night”; “After waking up in the night, the child has difficulty to fall asleep again”. All items were scored on a five-point Likert scale. Items were summed to create the sum score; the total score had an internal consistency of α = 0.63, with higher scores indicating more sleep problems. Both child- and mother-reported sleep problems were positively skewed.

#### Covariates

To address potential confounding, sex, age at assessment, child national origin, gestational age at birth, maternal education and maternal psychopathology were included as covariates. Child national origin was based on the birth country of the parents and categorized as Western (American Western, Asian Western, Dutch, European, Indonesian & Oceania) and Non-Western (Moroccan, Surinamese & Turkish, Dutch Antilles, African, American Non-Western, Asian Non-Western, Cape Verdean). Gestational age was entered in the model, because in the actigraphy subsample we oversampled children with a lower gestational age at birth [30]. Maternal education was assessed through questionnaire at birth and divided into three categories, being low (no education, primary school), middle (high school, vocational training), and high (higher vocational training, university). Maternal psychopathology was included using measures of the anxiety and depression subscales of the brief symptom inventory (BSI), which was completed when the child was 9 years of age [38].

### Statistical analyses

We assessed the cross-sectional relation between self- and mother-reported sleep problems and parent-reported mental health problems (internalizing, externalizing, dysregulation profile) at age 10–11 and 13–14. Linear regression analyses were used with sleep problems as the dependent and mental health problems as independent variables in separate models. Similarly, we analyzed the association between actigraphic sleep patterns (sleep duration, sleep onset, sleep onset latency, sleep efficiency, nocturnal awakenings) mental health problems. Lastly, we assess the relationship between the 24 h activity rhythm (IS, IV, L5 onset), and mental health problems.

All analyses were adjusted for different covariates in two step-wise models. The first model was adjusted for age and sex. The second model was additionally adjusted for gestational age at birth, child national origin, maternal educational level, and maternal psychopathology.

All analyses were performed in R 3.6.3 [39]. Our analyses include separate models for ten measures of sleep and three domains of mental health problems, performed at two different ages, resulting in a total of 60 tests. Because of the high number of models we adjusted for multiple testing using the Benjamini-Hochberg procedure [40]. Missing values on covariates, with a maximum of 6.1% missing, were imputed with multiple imputation through chained equations, using the mice package [41] (30 imputed datasets with 30 iterations).

#### Sensitivity and exploratory analyses

To assess the robustness of our findings, we performed two sensitivity analyses. First, as the actigraphy subsample might be affected by selection bias, we reran the analyses of self-reported sleep problems and mental health within the larger full samples of children that have self-reported sleep measures and CBCL data (10–11 years: n = 4180; 13–14 years: n = 3468). Second, we assessed whether individual domains of mental health underlie the associations between sleep measures (sleep patterns, 24 h activity rhythm and reported sleep) and the broad domains of mental health. The six syndrome scales that were used to calculate sum scores for the broad domains (anxious/depressed, withdrawn/depressed, somatic complaints, attention problems, rule-breaking behavior and aggressive behavior) were entered into separate models and analyzed using the same models and covariates used in the primary analyses.

To explore whether sleep problems and patterns are related to the two syndrome scales not included in the broad domains (social problems and thought problems), these syndrome scales were entered as independent variables in separate models as exploratory analyses.

## Results

### Sample characteristics

Table 1 shows the sample characteristics and mean scores for sleep measures and mental health problems of the children that participated in the study in the 10–11-year-old and the 13–14-year-old wave. Comparison of the two age groups revealed that apart from age, differences were present in maternal education, more mothers were classified as high education in the sample assessed at age 10–11. No significant differences were observed in the other demographic variables. Children in the 13–14-year-old wave had a shorter sleep duration, later sleep onset, shorter sleep onset latency, higher sleep efficiency, lower nocturnal waking times, a lower interdaily stability and intradaily variability, but no differences were present between the L5-onsets. Moreover, children in the 13–14-year-old wave had fewer self-reported sleep problems, but more internalizing symptoms.

**Table 1:** Sample characteristics

	10–11 years		13–14 years	
	n	788	n	344
Age actigraphy (M, SD)*	788	11.67 (0.20)	344	14.70 (0.33)
Age self-reported sleep (M, SD)*	788	9.75 (0.23)	344	13.93 (0.62)
Age CBCL (M, SD)*	788	9.70 (0.24)	344	13.52 (0.29)
Sex (%)				
Girl	414	52.5%	190	55.2%
Boy	374	47.5%	154	44.8%
National origin (%)				
Western	716	90.9%	318	92.4%
Non-Western	72	9.1%	26	7.6%
Maternal education level (%)*				
High	509	64.6%	194	56.4%
Middle	245	31.1%	139	40.4%
Low	10	1.3%	7	2.0%
Missing	24	4.7%	4	2.1%
Gestational age at birth (M, SD)	788	39.63 (2.26)	342	39.54 (2.31)
Maternal anxiety (M, SD)	773	1.43 (2.09)	323	1.39 (1.99)
Maternal depression (M, SD)	773	1.0 (2.16)	323	1.05 (2.02)
Behavior (M, SD)				
Internalizing*	788	4.47 (4.79)	344	5.25 (5.57)
Externalizing	788	3.59 (4.45)	344	3.63 (4.39)
Dysregulation profile	788	7.85 (7.27)	344	7.21 (7.55)
Sleep (M, SD)				
Sleep duration (time duration; hh:mm)*	788	7:42 (0:40)	344	7:16 (0:52)
Sleep onset (clock time; hh:mm)*	788	22:26 (0:54)	344	23:27 (0:50)
Sleep onset latency (time duration; hh:mm)*	788	0:52 (0:37)	344	0:19 (0:23)
Sleep efficiency (%)*	788	83.96 (4.52)	344	85.35 (5.15)
Nocturnal waking times*	786	3.05 (1.53)	344	2.83 (1.55)
Interdaily stability*	785	0.18 (0.04)	344	0.15 (0.05)
Intradaily variability*	785	0.58 (0.09)	344	0.52 (0.11)
Onset least active 5 h	786	00:47 (0:51)	344	00:44 (0:58)
Self-report*	788	2.50 (1.24)**	344	1.44 (1.26)
Mother-report	782	9.26 (2.64)	339	9.14 (2.65)

*Represent significant difference between the two age groups

**Range was different for both measurement waves, due to the use of different instruments, for ease of comparison this measurement was rescaled to range from 0 to 6

### Reported sleep problems

**Fig. 2:**
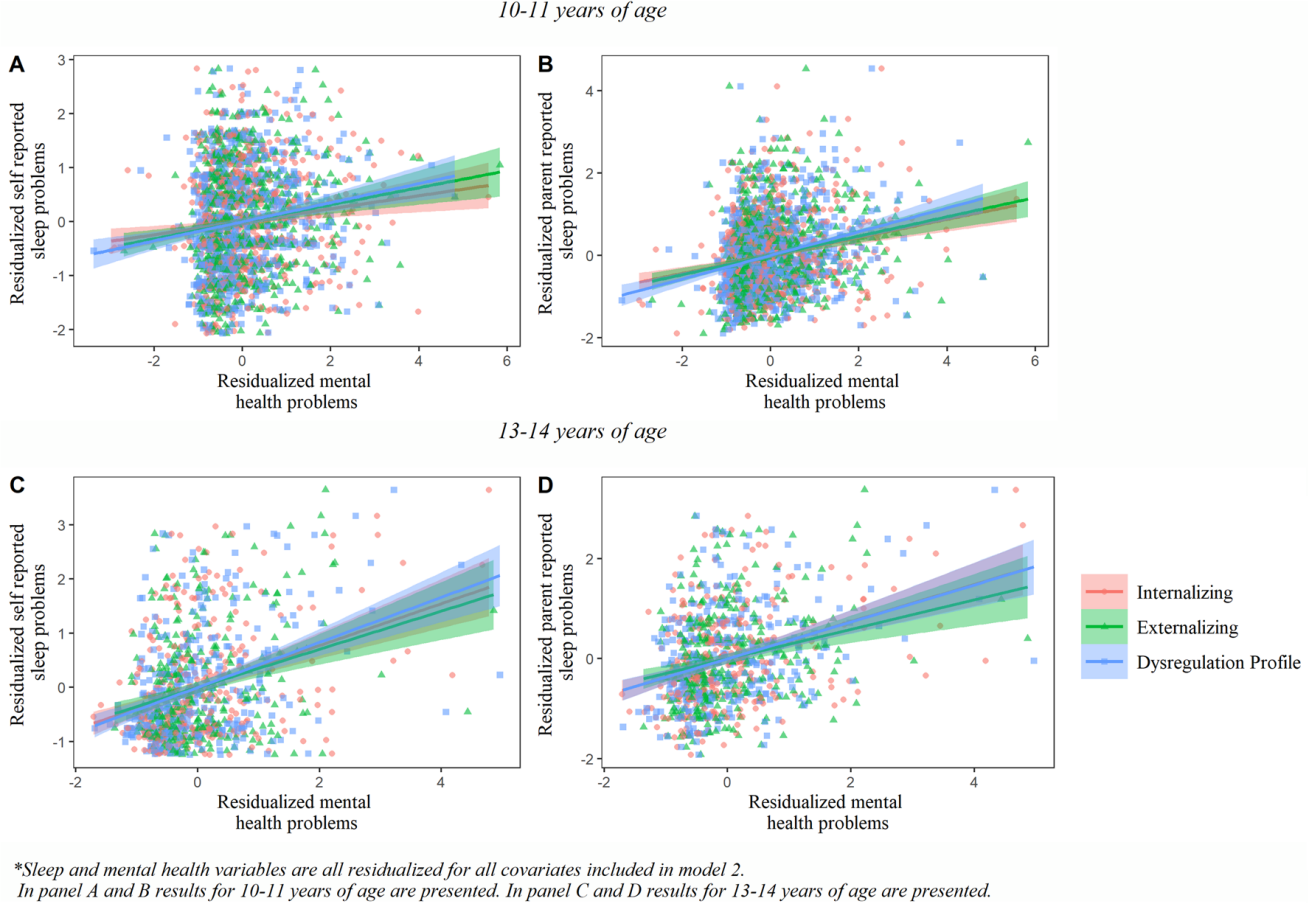
Associations of mental health problems with reported sleep problems

Associations between reported sleep problems and internalizing, externalizing, and dysregulation profile symptoms are presented in Table 2 and Fig. 2. Positive associations were observed between both mother-reported and self-reported sleep problems and internalizing symptoms (range standardized ß-estimates: 0.11–0.35), externalizing symptoms (range standardized ß-estimates: 0.13–0.24), and dysregulation profile symptoms (range standardized ß-estimates: 0.14–0.33). These relationships were present after adjustment for sex, age, gestational age at birth, child national origin, maternal education and maternal psychopathology. All results remained after correction for multiple testing.

**Table 2:** Associations of mental health problems with reported sleep problems

Sleep problems	Model	ß	SE	p-value	ß	SE	p-value
		10–11 years			13–14 years		
		*Internalizing problems*					
Self-reported	1	0.12	0.03	0.001	0.31	0.05	< 0.001
	2	0.11	0.03	0.002*	0.31	0.05	< 0.001*
Mother-reported	1	0.25	0.03	< 0.001	0.35	0.05	< 0.001
	2	0.20	0.03	< 0.001*	0.34	0.05	< 0.001*
		*Externalizing problems*					
Self-reported	1	0.14	0.03	< 0.001	0.22	0.04	< 0.001
	2	0.13	0.03	< 0.001*	0.22	0.04	< 0.001*
Mother-reported	1	0.24	0.03	< 0.001	0.21	0.04	< 0.001
	2	0.20	0.03	< 0.001*	0.20	0.04	< 0.001*
		*Dysregulation profile*					
Self-reported	1	0.16	0.03	< 0.001	0.32	0.05	< 0.001
	2	0.14	0.03	< 0.001*	0.32	0.05	< 0.001*
Mother-reported	1	0.29	0.03	< 0.001	0.33	0.05	< 0.001
	2	0.24	0.03	<0.001*	0.32	0.05	< 0.001*

Sleep characteristics and behavioral measurements were standardized to a mean of 0 and SD 1, ß coefficients represent change in SD

Model 1 is corrected for age at reported sleep measurement, age at behavioral measurement and sex, model 2 is additionally corrected for gestational age at birth, child national origin, maternal education and maternal psychopathology

*Significant after FDR-BH correction for multiple testing

### Actigraphy estimated sleep and 24-h activity rhythms

**Fig. 3:**
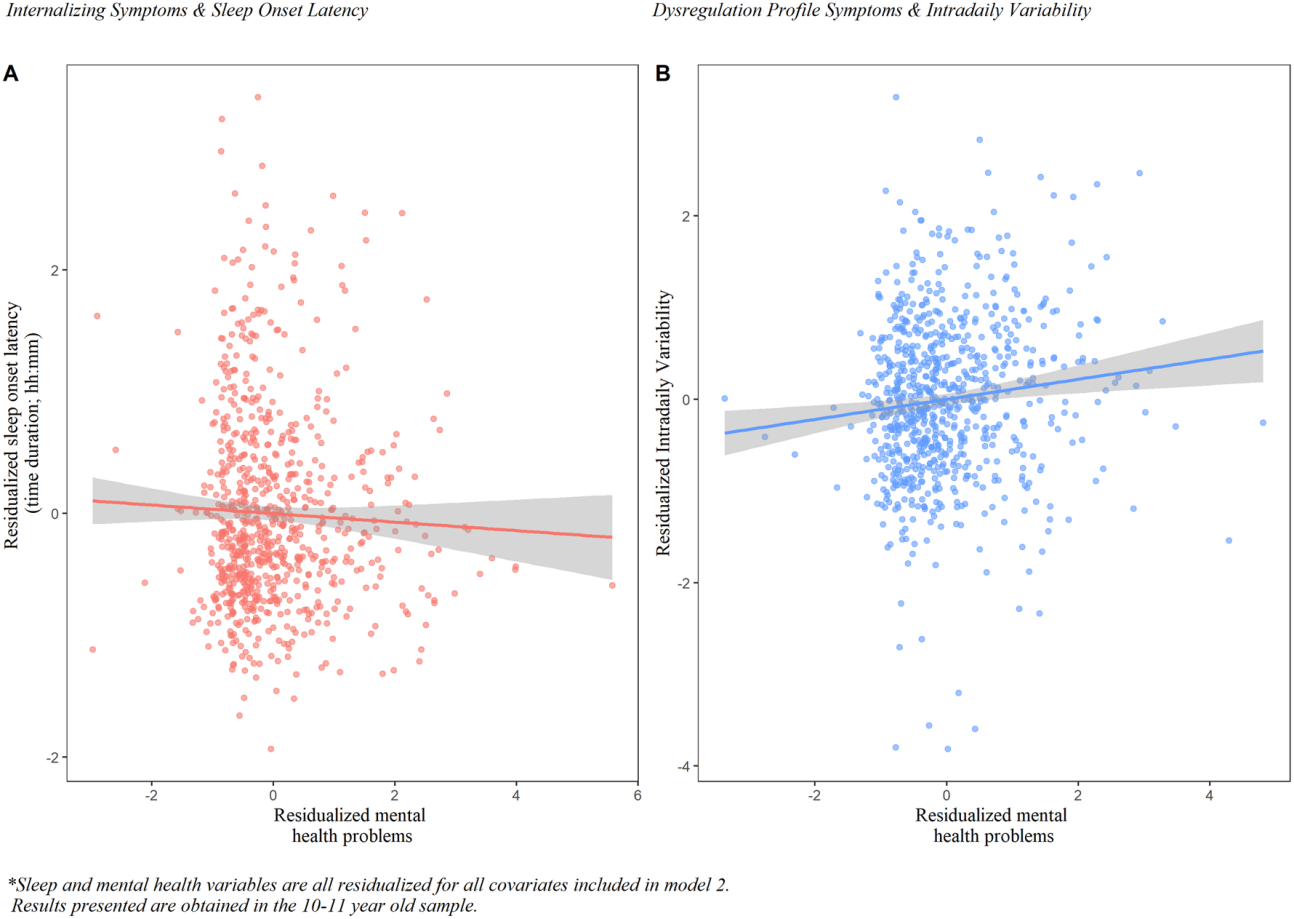
Significant associations of mental health problems and sleep measured by actigraphy

Results of the cross-sectional relation between actigraphic sleep patterns and symptoms of mental health problems are presented in Table 3 and Fig. 3. Earlier sleep onset was associated with more internalizing problems in 10–11-year-old children (ß = 0.09, SE = 0.03, p-value = 0.002). No associations between sleep duration, sleep onset latency, sleep efficiency, and nocturnal waking times and any of the mental health domains were observed.

**Table 3:** Associations of mental health problems with actigraphic sleep patterns

Sleep patterns	Model	ß	SE	p-value	ß	SE	p-value
		10–11 years			13–14 years		
		*Internalizing problems*					
Sleep duration	1	0.013	0.030	0.657	0.039	0.049	0.423
	2	0.034	0.031	0.279	0.054	0.050	0.285
Sleep onset	1	− 0.063	0.027	0.021	− 0.068	0.041	0.095
	2	− 0.087	0.028	0.002*	− 0.072	0.042	0.086
Sleep onset latency	1	− 0.030	0.030	0.322	− 0.018	0.023	0.436
	2	− 0.036	0.032	0.254	− 0.030	0.024	0.208
Sleep efficiency	1	0.016	0.029	0.577	− 0.105	0.051	0.038
	2	0.011	0.031	0.712	− 0.091	0.052	0.083
Nocturnal waking times	1	− 0.024	0.035	0.494	0.100	0.055	0.067
	2	− 0.006	0.037	0.864	0.103	0.056	0.067
		*Externalizing problems*					
Sleep duration	1	− 0.014	0.032	0.650	0.033	0.057	0.567
	2	0.005	0.032	0.876	0.042	0.058	0.477
Sleep onset	1	− 0.008	0.029	0.772	0.000	0.048	0.997
	2	− 0.027	0.029	0.352	− 0.014	0.049	0.771
Sleep onset latency	1	0.006	0.032	0.863	− 0.036	0.027	0.185
	2	0.003	0.033	0.939	− 0.050	0.027	0.070
Sleep efficiency	1	0.014	0.031	0.656	− 0.002	0.059	0.979
	2	0.014	0.032	0.664	− 0.006	0.061	0.921
Nocturnal waking times	1	− 0.029	0.037	0.434	0.010	0.064	0.881
	2	− 0.018	0.039	0.648	0.020	0.065	0.757
		*Dysregulation profile*					
Sleep duration	1	0.004	0.031	0.910	0.074	0.050	0.141
	2	0.022	0.033	0.504	0.086	0.052	0.095
Sleep onset	1	− 0.040	0.028	0.155	− 0.056	0.042	0.183
	2	− 0.063	0.030	0.034	− 0.064	0.043	0.138
Sleep onset latency	1	0.008	0.031	0.801	−0.037	0.024	0.128
	2	0.011	0.033	0.743	− 0.048	0.024	0.048
Sleep efficiency	1	0.020	0.030	0.505	− 0.049	0.053	0.350
	2	0.017	0.032	0.593	− 0.041	0.054	0.445
Nocturnal waking times	1	− 0.038	0.037	0.298	0.058	0.057	0.312
	2	− 0.024	0.039	0.533	0.063	0.058	0.275

Sleep characteristics and behavioral measurements were standardized to a mean of 0 and SD 1, ß coefficients represent change in SD

Model 1 is corrected for age at actigraphy measurement, age at behavioral measurement and sex, model 2 is additionally corrected for gestational age at birth, child national origin, maternal education and maternal psychopathology

*Significant after FDR-BH correction for multiple testing

Table 4 shows the relationship between 24 h activity rhythms and mental health problems. We observed that a higher intradaily variability was statistically significant associated with more dysregulation profile symptoms in our 10–11-year-old age group (ß = 0.11, SE = 0.04 p-value = 0.002) (Table 4). However, these relationships were not found in the 13–14-year-old children and no other relationships were observed between the 24 h activity rhythm and mental health problems.

**Table 4:** Associations of mental health problems and the 24 h activity rhythm, measured by actigraphy

24 h activity rhythm	Model	ß	SE	p-value	ß	SE	p-value
		10–11 years			13–14 years		
		*Internalizing problems*					
Interdaily stability	1	− 0.012	0.030	0.693	0.032	0.050	0.527
	2	− 0.007	0.032	0.835	− 0.003	0.051	0.946
Intradaily variability	1	0.076	0.033	0.020	0.077	0.061	0.206
	2	0.079	0.034	0.022	0.070	0.063	0.266
Onset least active 5 h	1	− 0.059	0.033	0.078	− 0.035	0.055	0.526
	2	− 0.054	0.035	0.120	− 0.019	0.056	0.735
		*Externalizing problems*					
Interdaily stability	1	0.024	0.032	0.446	0.048	0.059	0.415
	2	0.033	0.033	0.305	0.029	0.059	0.631
Intradaily variability	1	0.022	0.035	0.533	0.003	0.071	0.969
	2	0.016	0.036	0.661	− 0.008	0.072	0.913
Onset least active 5 h	1	− 0.001	0.035	0.967	− 0.056	0.064	0.386
	2	0.002	0.036	0.951	− 0.051	0.065	0.432
		*Dysregulation profile*					
Interdaily stability	1	0.025	0.031	0.424	0.051	0.052	0.324
	2	0.040	0.033	0.229	0.027	0.053	0.610
Intradaily variability	1	0.106	0.034	0.002*	0.085	0.063	0.177
	2	0.109	0.036	0.002*	0.080	0.064	0.215
Onset least active 5 h	1	− 0.039	0.035	0.269	− 0.055	0.057	0.336
	2	− 0.028	0.037	0.446	− 0.047	0.058	0.415

Sleep characteristics and behavioral measurements were standardized to a mean of 0 and SD 1, ß coefficients represent change in SD

Model 1 is corrected for age at actigraphy measurement, age at behavioral measurement and sex, model 2 is additionally corrected for gestational age at birth, child national origin, maternal education and maternal psychopathology

*Significant after FDR-BH correction for multiple testing

### Sensitivity and exploratory analyses

First, we assessed whether the findings on self-reported sleep problems and mental health problems were similar in the full sample of Generation R participants and in the actigraphic subsample. No substantial differences were observed, similar to our primary findings, self-reported sleep problems were strongly associated with all domains of mental health problems in the larger sample (Additional file 1: Table S1).

Second, we assessed whether specific syndrome scales accounted for the associations between sleep measures and the broad domains of mental health that we studied. Reported sleep problems were associated with all syndrome scales, except for the withdrawn/depressed syndrome scale at 10–11 years (Additional file 1: Table S2). For actigraphy-estimated sleep, associations were observed between sleep onset, sleep onset latency, sleep efficiency and nocturnal awakenings and the syndrome scales included in the internalizing domain (anxiety/depressed, withdrawn/depressed and somatic complaints) (Additional file 1: Table S3). For the 24 h activity rhythm, we found that the relationship observed between intradaily variability and dysregulation profile symptoms in our 10–11-year-old wave was mainly driven by attention problems (ß = 0.165, p-value = 2.22 × 10^−6^ (Additional file 1: Table S4).

In our exploratory analyses, we analyzed those syndrome scales that were not included in the broad domains of mental health problems. Reported sleep problems were positively associated with both social problems and thought problems (Additional file 1: Table S5). Regarding actigraphic sleep measures, a longer sleep duration ((ß = 0.09, p-value = 0.009), earlier sleep onset (ß = − 0.11, p-value = 2.64 × 10^−4^) and a higher intradaily variability (ß = 0.12, p-value = 0.001) were related to more social problems at 10–11 years (Additional file 1: Tables S6, S7).

## Discussion

This population-based study provides evidence for associations between sleep problems reported by different informants and mental health problems in late childhood and early adolescence. We observed that more reported sleep problems were related to more mental health problems, which was consistent across reporters, different sleep questionnaires and all domains of psychopathology, bolstering the robustness of the associations detected. Notably, these findings are in line with the existing literature in adults [6, 42, 43]. Contrary to our hypotheses, we did not observe any associations between actigraphic sleep patterns and broad domains of mental health apart from an earlier sleep onset in those with more internalizing symptoms. Regarding the 24 h activity rhythm measures, we observed that a higher intradaily variability of the 24 h activity rhythm was related to more dysregulation profile symptoms at 10–11 years of age. This association did not reach significance at 13–14 years of age, but the effect size was similar. No other associations between mental health problems and the 24 h activity rhythm were observed.

We found a notable discrepancy between results from reported and actigraphic sleep measures. This discrepancy was present across all broad domains and most individual syndrome scales of mental health problems. Although earlier work in children with dysregulation symptoms, ADHD, and depression showed reduced sleep efficiency using both reported and objective sleep measures [9, 14, 18], we did not observe associations between decreased sleep efficiency and the corresponding behavioral scales in our study. Likewise, we did not replicate earlier work from clinical populations that showed a shorter sleep duration in children with ADHD [14] or longer sleep duration in children with depression [10]. However, our findings were in line with previous actigraphic studies that reported no associations between continuous ADHD symptoms and sleep parameters [44]. Similarly, in previous population-based samples actigraphic sleep parameters were not associated with depressive and anxiety symptoms [45]. Multiple explanations can underlie the discrepancy between reported and actigraphic sleep measures. First, sleep is multi-facetted and it is possible that actigraphic sleep measures, such as total sleep time are unaffected, while the neuronal activity during sleep in children with mental health problems is affected. Possibly, reported sleep taps into those domains of sleep that are not captured by actigraphy, neuronal activities such as arousal and sleep depth. This possibility is supported by the general absence of significant correlations between reported and actigraphic sleep measures (Additional file 1: Figs. S1, S2). Second, actigraphic sleep measures were obtained approximately 1 year after assessment of mental health. Even though there is considerable continuity of mental health problems between late childhood and early adolescence [46], the effect may have attenuated due to the time interval between measurements. Third, it is possible that there truly is no relationship between objective sleep measures and most domains of mental health problems in a population-based setting, but that children and adolescents with more psychopathology are simply more likely to report sleep problems. This would imply that asking children about their sleep is very relevant for detecting co-occurring mental health problems, but we do not provide evidence that assessing their sleep using objective sleep measures is equally relevant to detect co-occurring mental health problems. It is important to address the relation between sleep and mental health in future work with PSG to clarify the discrepancy between reported and actigraphic sleep measures. PSG is able to capture the neuronal activity during sleep that potentially map more closely to those assessed with reported sleep.

We observed a relationship between intradaily variability and dysregulation symptoms in our 10–11-year-old sample. The effect size was similar in the 13–14-year-old sample, although it did not reach statistical significance, potentially because of the smaller sample size. Our sensitivity analyses, where we analyzed syndrome scales separately, revealed that this association was largely driven by the relationship with attention problems and to a small extent by anxiety/depression, but not by aggressive behavior. This indicates that not children with the dysregulation profile have a more fragmented pattern of active and rest states, but rather those with attention problems. In line with these findings, a delayed 24 h activity rhythm has been proposed to be partly underlying ADHD symptoms [47]. Longitudinal research in clinical samples is needed to unravel how ADHD and the 24 h activity rhythm influence each other over time.

Our findings have both clinical and future research implications. Given the associations between reported sleep measures and all domains of mental health problems, the current study and earlier work suggest that sleep problems might have prognostic value for detecting general mental health problems [1]. Potentially, talking about sleep problems can help caregivers identify emerging mental health problems and seek appropriate help in an early stage. However, to adequately address to what extent sleep problems can indeed be used for this purpose, longitudinal studies delineating the causal pathway between sleep and mental health problems are needed. This is especially crucial for a better understanding of the relationship between sleep and mental health, given that the relationship is likely to be bidirectional, meaning that sleep difficulties can potentially preceed mental health problems as well as vice versa. Second, in our multi-method approach, we identified discrepancies between results for reported sleep measures and actigraphic sleep characteristics. Often, reported and actigraphic sleep measures are considered to be respectively subjective and objective sleep measures, suggesting they are different measures of the same underlying construct [48]. However, the current results, together with earlier findings within the current sample showing low correlations between reported and actigraphic sleep measures [32], show that they may tap into distinct constructs. Indeed, this is in line with other studies incorporating both reported and actigraphic sleep measures [49]. Reported sleep is a measure of perceived sleep quality [50], whereas actigraphic sleep is derived from activity measured by an accelerometer [51]. Potentially, actigraphic sleep is a more physiological measure particularly relevant for understanding cardiovascular and genetic factors [52], whereas the perception of sleep problems is a better indicator of mental health outcomes [1].

Our findings should be considered in the light of some limitations. First, we used questionnaires to identify child mental health problems, it would have been optimal to have also obtained clinical psychiatric interviews. Second, we used different questionnaires to assess self-reported sleep at age 10–11 and at age 13–14 years. Notwithstanding these differences, results were similar across ages. Third, while questionnaires regarding behavior were focused on the past 6 months, no time frame was given to children to report on their perceived sleep problems. Fourth, in our analyses between mother-reported sleep problems and mother-reported mental health problems, estimates might be inflated due to reporter bias. However, results were consistent with self-reported sleep problems. Fifth, the current study was cross-sectional and therefore unable to address the temporal relationship between sleep and mental health problems. Lastly, even though we had a large sample size, given the relatively low number of children that have mental health problems and altered sleep patterns or 24 h activity rhythm, it might be that some relationships remained undetected due to lack of power. The current study had several strengths. We studied a large population-based sample, in which both actigraphic and reported sleep problems were measured as well as a broad spectrum of mental health problems and confounders, enabling us to directly compare actigraphic and reported sleep measures and explore patterns of associations across domains of mental health. Moreover, we included children both during late childhood and in early adolescence, which has shown to be a vulnerable period for development of sleep problems and psychopathology [3–5].

## Conclusions

In conclusion, this large study with multimodal assessments of sleep provided evidence for consistent associations between mental health problems and reported sleep problems in a large population-based sample of children and adolescents. The results underscore the role of perceived sleep problems in children with mental health problems across all domains. However, we found few associations between actigraphic sleep patterns and 24 h activity rhythm measures and mental health problems. The discrepancies between the reported and actigraphic sleep measures strengthen the idea that these sleep measures assess different constructs of sleep, rather than providing a subjective and an objective measure of the same underlying construct. A promising extension of our findings regarding reported sleep would be to use longitudinal data to assess to what extent the relationship between mental health problems and reported sleep problems is bidirectional. Regarding objective sleep measures, more work is needed to confirm the absence of the relationship between mental health problems and actigraphic sleep patterns in samples enriched for mental health problems. Additionally, the use of PSG is important to provide more insight in sleep in individuals with mental health problems.

## Supplementary Information

**Additional file 1.** Supplemental methods and results, including results from sensitivity analyses and exploratory analyses.

## Data Availability

Generation R data is available to researchers upon reasonable request. Request should be directed to the management team of the Generation R Study. Individual level data are not publicly available for privacy and ethical restrictions. The code is available upon request from the corresponding author.
