# Supplementary material for: Sleep and mental health in childhood: a multi-method study in the general pediatric population
Source: Child Adolesc Psychiatry Ment Health. 2022 Feb 17;16:11. doi: 10.1186/s13034-022-00447-0 (PMC8851725; doi:10.1186/s13034-022-00447-0)
Supplement: Supplementary file 1 — Additional file 1. Supplemental methods and results, including results from sensitivity analyses and exploratory analyses. [file 13034_2022_447_MOESM1_ESM.docx]

**Additional material**

*Additional methods and results*

Nocturnal waking times

As supplementary follow-up analyses, we reran all models including the variable nocturnal waking times as a count variable. To this end, we applied negative binomial regression analyses in which we used the untransformed variable for nocturnal waking times as the dependent, and mental health problems as the independent variables. Consistent with our main analyses, we performed these analyses using two models. In model 1 we corrected for age at reported sleep measurement, age at behavioral measurement and sex, and in model 2 we additionally corrected for gestational age at birth, child national origin, maternal education and maternal psychopathology. Results obtained from these models were highly similar to those from our main analyses, in which no statistically significant associations were observed between nocturnal waking times and mental health problems. Full results are shown in Table S8.

*Additional Tables*

| **Table S1. Associations of mental health problems with reported sleep problems in the full Generation R sample** | | | | | | | | |
| --- | --- | --- | --- | --- | --- | --- | --- | --- |
|  | model | ß | SE | p-value |  | ß | SE | p-value |
|  |  | 10-11 years | | |  | 13-14 years | | |
|  |  | *Internalizing problems* | | | | | | |
| Self-reported | 1 | 0.185 | 0.015 | <0.001 |  | 0.260 | 0.016 | <0.001 |
|  | 2 | 0.164 | 0.014 | <0.001 |  | 0.246 | 0.016 | <0.001 |
| Parent-reported | 1 | 0.226 | 0.027 | <0.001 |  | 0.289 | 0.029 | <0.001 |
|  | 2 | 0.192 | 0.027 | <0.001 |  | 0.263 | 0.029 | <0.001 |
|  |  | *Externalizing problems* | | | | | | |
| Self-reported | 1 | 0.166 | 0.015 | <0.001 |  | 0.200 | 0.015 | <0.001 |
|  | 2 | 0.151 | 0.014 | <0.001 |  | 0.191 | 0.015 | <0.001 |
| Parent-reported | 1 | 0.184 | 0.026 | <0.001 |  | 0.220 | 0.028 | <0.001 |
|  | 2 | 0.157 | 0.026 | <0.001 |  | 0.202 | 0.028 | <0.001 |
|  |  | *Dysregulation profile* | | | | | | |
| Self-reported | 1 | 0.212 | 0.015 | <0.001 |  | 0.250 | 0.016 | <0.001 |
|  | 2 | 0.192 | 0.014 | <0.001 |  | 0.239 | 0.016 | <0.001 |
| Parent-reported | 1 | 0.247 | 0.026 | <0.001 |  | 0.314 | 0.029 | <0.001 |
|  | 2 | 0.214 | 0.026 | <0.001 |  | 0.292 | 0.030 | <0.001 |
| Sleep characteristics and behavioral measurements were standardized to a mean of 0 and SD 1, ß coefficients represent change in SD. | | | | | | | | |
| Model 1 is corrected for age at reported sleep measurement, age at behavioral measurement and sex, model 2 is additionally corrected for gestational age at birth, child national origin, maternal education and maternal psychopathology | | | | | | | | |

| **Table S2. Sensitivity analyses: associations of individual syndrome scales with reported sleep problems** | | | | | | | | |
| --- | --- | --- | --- | --- | --- | --- | --- | --- |
|  | model | ß | SE | p-value |  | ß | SE | p-value |
|  |  | 10-11 years | | |  | 13-14 years | | |
|  |  | *Anxiety/Depression* | | | | | | |
| Self-reported | 1 | 0.117 | 0.036 | 0.001 |  | 0.254 | 0.052 | <0.001 |
|  | 2 | 0.099 | 0.035 | 0.004 |  | 0.255 | 0.052 | <0.001 |
| Parent-reported | 1 | 0.260 | 0.035 | <0.001 |  | 0.279 | 0.056 | <0.001 |
|  | 2 | 0.217 | 0.035 | <0.001 |  | 0.274 | 0.055 | <0.001 |
|  |  | *Withdrawn/Depressed* | | | | | | |
| Self-reported | 1 | 0.038 | 0.036 | 0.285 |  | 0.272 | 0.046 | <0.001 |
|  | 2 | 0.023 | 0.035 | 0.505 |  | 0.277 | 0.046 | <0.001 |
| Parent-reported | 1 | 0.168 | 0.036 | <0.001 |  | 0.333 | 0.048 | <0.001 |
|  | 2 | 0.135 | 0.036 | <0.001 |  | 0.318 | 0.049 | <0.001 |
|  |  | *Somatic Complaints* | | | | | | |
| Self-reported | 1 | 0.122 | 0.033 | <0.001 |  | 0.199 | 0.044 | <0.001 |
|  | 2 | 0.113 | 0.032 | 0.001 |  | 0.206 | 0.043 | <0.001 |
| Parent-reported | 1 | 0.148 | 0.033 | <0.001 |  | 0.239 | 0.045 | <0.001 |
|  | 2 | 0.113 | 0.034 | 0.001 |  | 0.223 | 0.045 | <0.001 |
|  |  | *Attention Problems* | | | | | | |
| Self-reported | 1 | 0.112 | 0.034 | 0.001 |  | 0.319 | 0.051 | <0.001 |
|  | 2 | 0.098 | 0.033 | 0.003 |  | 0.323 | 0.051 | <0.001 |
| Parent-reported | 1 | 0.213 | 0.034 | <0.001 |  | 0.325 | 0.053 | <0.001 |
|  | 2 | 0.174 | 0.034 | <0.001 |  | 0.316 | 0.054 | <0.001 |
|  |  | *Rule-breaking Behavior* | | | | | | |
| Self-reported | 1 | 0.079 | 0.033 | 0.017 |  | 0.166 | 0.040 | <0.001 |
|  | 2 | 0.070 | 0.033 | 0.033 |  | 0.166 | 0.040 | <0.001 |
| Parent-reported | 1 | 0.162 | 0.033 | <0.001 |  | 0.166 | 0.043 | <0.001 |
|  | 2 | 0.132 | 0.034 | <0.001 |  | 0.157 | 0.043 | <0.001 |
|  |  | *Aggressive Behavior* | | | | | | |
| Self-reported | 1 | 0.155 | 0.033 | <0.001 |  | 0.223 | 0.043 | <0.001 |
|  | 2 | 0.142 | 0.033 | <0.001 |  | 0.221 | 0.043 | <0.001 |
| Parent-reported | 1 | 0.243 | 0.033 | <0.001 |  | 0.214 | 0.046 | <0.001 |
|  | 2 | 0.211 | 0.033 | <0.001 |  | 0.203 | 0.046 | <0.001 |
| Sleep characteristics and behavioral measurements were standardized to a mean of 0 and SD 1, ß coefficients represent change in SD. | | | | | | | | |
| Model 1 is corrected for age at reported sleep measurement, age at behavioral measurement and sex, model 2 is additionally corrected for gestational age at birth, child national origin, maternal education and maternal psychopathology | | | | | | | | |

| **Table S3. Sensitivity analyses: associations of individual syndrome scales with actigraphic sleep patterns** | | | | | | | | |
| --- | --- | --- | --- | --- | --- | --- | --- | --- |
|  | model | ß | SE | p-value |  | ß | SE | p-value |
|  |  | 10-11 years | | |  | 13-14 years | | |
|  |  | *Anxiety/Depression* | | | | | | |
| Sleep duration | 1 | 0.001 | 0.029 | 0.974 |  | 0.071 | 0.045 | 0.117 |
|  | 2 | 0.013 | 0.030 | 0.660 |  | 0.029 | 0.017 | 0.088 |
| Sleep onset | 1 | -0.045 | 0.027 | 0.087 |  | -0.082 | 0.038 | 0.031 |
|  | 2 | -0.063 | 0.027 | 0.023 |  | -0.030 | 0.014 | 0.033 |
| Nocturnal waking times | 1 | -0.011 | 0.035 | 0.760 |  | 0.060 | 0.051 | 0.239 |
|  | 2 | 0.008 | 0.036 | 0.819 |  | 0.022 | 0.019 | 0.239 |
|  |  | *Withdrawn/Depressed* | | | | | | |
| Sleep duration | 1 | 0.021 | 0.029 | 0.473 |  | 0.015 | 0.023 | 0.512 |
|  | 2 | 0.034 | 0.030 | 0.259 |  | 0.021 | 0.024 | 0.372 |
| Sleep onset | 1 | -0.056 | 0.027 | 0.035 |  | -0.005 | 0.019 | 0.798 |
|  | 2 | -0.069 | 0.027 | 0.011 |  | -0.006 | 0.020 | 0.775 |
| Sleep onset latency | 1 | -0.016 | 0.029 | 0.576 |  | 0.004 | 0.011 | 0.720 |
|  | 2 | -0.018 | 0.030 | 0.559 |  | <0.001 | 0.011 | 0.970 |
| Sleep efficiency | 1 | 0.039 | 0.028 | 0.174 |  | -0.005 | 0.024 | 0.839 |
|  | 2 | 0.038 | 0.029 | 0.190 |  | 0.001 | 0.025 | 0.978 |
| Nocturnal waking times | 1 | -0.060 | 0.035 | 0.084 |  | 0.003 | 0.026 | 0.917 |
|  | 2 | -0.051 | 0.036 | 0.157 |  | 0.001 | 0.027 | 0.962 |
|  |  | *Somatic Complaints* | | | | | | |
| Sleep duration | 1 | 0.018 | 0.032 | 0.570 |  | -0.011 | 0.024 | 0.663 |
|  | 2 | 0.042 | 0.032 | 0.199 |  | -0.004 | 0.025 | 0.880 |
| Sleep onset | 1 | -0.054 | 0.029 | 0.061 |  | -0.030 | 0.021 | 0.148 |
|  | 2 | -0.077 | 0.029 | 0.009 |  | -0.032 | 0.021 | 0.133 |
| Sleep onset latency | 1 | -0.047 | 0.032 | 0.140 |  | -0.004 | 0.012 | 0.742 |
|  | 2 | -0.056 | 0.033 | 0.089 |  | -0.009 | 0.012 | 0.447 |
| Sleep efficiency | 1 | -0.001 | 0.031 | 0.980 |  | -0.083 | 0.025 | 0.001 |
|  | 2 | <0.001 | 0.032 | 0.991 |  | -0.078 | 0.026 | 0.003 |
| Nocturnal waking times | 1 | 0.004 | 0.038 | 0.913 |  | 0.092 | 0.027 | 0.001 |
|  | 2 | 0.016 | 0.039 | 0.670 |  | 0.095 | 0.028 | 0.001 |
|  |  | *Attention Problems* | | | | | | |
| Sleep duration | 1 | 0.019 | 0.031 | 0.528 |  | 0.012 | 0.014 | 0.391 |
|  | 2 | 0.031 | 0.032 | 0.326 |  | 0.015 | 0.015 | 0.302 |
| Sleep onset | 1 | -0.040 | 0.028 | 0.151 |  | -0.008 | 0.012 | 0.510 |
|  | 2 | -0.055 | 0.029 | 0.058 |  | -0.009 | 0.012 | 0.443 |
| Sleep onset latency | 1 | 0.025 | 0.031 | 0.423 |  | -0.004 | 0.007 | 0.549 |
|  | 2 | 0.036 | 0.032 | 0.268 |  | -0.005 | 0.007 | 0.425 |
| Sleep efficiency | 1 | 0.025 | 0.030 | 0.396 |  | -0.008 | 0.015 | 0.578 |
|  | 2 | 0.026 | 0.031 | 0.398 |  | -0.006 | 0.015 | 0.683 |
| Nocturnal waking times | 1 | -0.052 | 0.036 | 0.153 |  | 0.015 | 0.016 | 0.342 |
|  | 2 | -0.047 | 0.038 | 0.217 |  | 0.016 | 0.016 | 0.319 |
|  |  | *Rule-breaking Behavior* | | | | | | |
| Sleep duration | 1 | -0.020 | 0.031 | 0.523 |  | -0.028 | 0.033 | 0.403 |
|  | 2 | <0.001 | 0.032 | 0.999 |  | -0.025 | 0.034 | 0.459 |
| Sleep onset | 1 | 0.004 | 0.029 | 0.892 |  | 0.031 | 0.028 | 0.262 |
|  | 2 | -0.013 | 0.029 | 0.646 |  | 0.024 | 0.028 | 0.408 |
| Sleep onset latency | 1 | 0.005 | 0.032 | 0.881 |  | -0.010 | 0.016 | 0.542 |
|  | 2 | <0.001 | 0.032 | 0.988 |  | -0.018 | 0.016 | 0.263 |
| Sleep efficiency | 1 | 0.004 | 0.030 | 0.886 |  | 0.015 | 0.035 | 0.662 |
|  | 2 | 0.006 | 0.031 | 0.843 |  | 0.011 | 0.036 | 0.758 |
| Nocturnal waking times | 1 | -0.026 | 0.037 | 0.482 |  | -0.011 | 0.038 | 0.767 |
|  | 2 | -0.017 | 0.038 | 0.655 |  | -0.003 | 0.038 | 0.931 |
|  |  | *Aggressive Behavior* | | | | | | |
| Sleep duration | 1 | -0.011 | 0.031 | 0.735 |  | 0.016 | 0.014 | 0.257 |
|  | 2 | 0.006 | 0.032 | 0.845 |  | 0.019 | 0.015 | 0.202 |
| Sleep onset | 1 | -0.012 | 0.028 | 0.663 |  | -0.006 | 0.012 | 0.620 |
|  | 2 | -0.030 | 0.029 | 0.306 |  | -0.009 | 0.012 | 0.452 |
| Sleep onset latency | 1 | 0.005 | 0.031 | 0.871 |  | -0.010 | 0.007 | 0.129 |
|  | 2 | 0.003 | 0.032 | 0.919 |  | -0.013 | 0.007 | 0.052 |
| Sleep efficiency | 1 | 0.015 | 0.030 | 0.615 |  | -0.003 | 0.015 | 0.821 |
|  | 2 | 0.014 | 0.031 | 0.642 |  | -0.004 | 0.015 | 0.789 |
| Nocturnal waking times | 1 | -0.026 | 0.037 | 0.477 |  | 0.005 | 0.016 | 0.739 |
|  | 2 | -0.015 | 0.038 | 0.697 |  | 0.008 | 0.017 | 0.649 |
| Sleep characteristics and behavioral measurements were standardized to a mean of 0 and SD 1, ß coefficients represent change in SD. | | | | | | | | |
| Model 1 is corrected for age at actigraphy measurement, age at behavioral measurement and sex, model 2 is additionally corrected for gestational age at birth, child national origin, maternal education and maternal psychopathology | | | | | | | | |

| **Table S4. Sensitivity analyses: associations of individual syndrome scales and the 24h activity rhythm, measured by actigraphy** | | | | | | | | |
| --- | --- | --- | --- | --- | --- | --- | --- | --- |
|  | model | ß | SE | p-value |  | ß | SE | p-value |
|  |  | 10-11 years | | |  | 13-14 years | | |
|  |  | *Anxiety/Depression* | | | | | | |
| Interdaily Stability | 1 | 0.023 | 0.029 | 0.423 |  | 0.046 | 0.047 | 0.324 |
|  | 2 | 0.029 | 0.030 | 0.343 |  | 0.009 | 0.017 | 0.607 |
| Intradaily Variability | 1 | 0.069 | 0.032 | 0.032 |  | 0.114 | 0.056 | 0.045 |
|  | 2 | 0.072 | 0.033 | 0.031 |  | 0.038 | 0.021 | 0.067 |
| Onset least active 5 hours | 1 | -0.040 | 0.033 | 0.224 |  | -0.064 | 0.051 | 0.212 |
|  | 2 | -0.029 | 0.034 | 0.385 |  | -0.018 | 0.019 | 0.338 |
|  |  | *Withdrawn/Depressed* | | | | | | |
| Interdaily Stability | 1 | -0.016 | 0.029 | 0.588 |  | 0.001 | 0.024 | 0.969 |
|  | 2 | -0.011 | 0.030 | 0.723 |  | -0.014 | 0.024 | 0.558 |
| Intradaily Variability | 1 | 0.063 | 0.032 | 0.051 |  | 0.020 | 0.029 | 0.480 |
|  | 2 | 0.063 | 0.033 | 0.058 |  | 0.019 | 0.029 | 0.523 |
| Onset least active 5 hours | 1 | -0.071 | 0.033 | 0.031 |  | -0.010 | 0.026 | 0.713 |
|  | 2 | -0.067 | 0.033 | 0.045 |  | -0.006 | 0.026 | 0.832 |
|  |  | *Somatic Complaints* | | | | | | |
| Interdaily Stability | 1 | -0.056 | 0.032 | 0.076 |  | 0.007 | 0.025 | 0.788 |
|  | 2 | -0.053 | 0.033 | 0.105 |  | -0.011 | 0.026 | 0.666 |
| Intradaily Variability | 1 | 0.047 | 0.035 | 0.181 |  | 0.004 | 0.030 | 0.896 |
|  | 2 | 0.041 | 0.036 | 0.250 |  | -0.001 | 0.032 | 0.964 |
| Onset least active 5 hours | 1 | -0.035 | 0.035 | 0.321 |  | 0.008 | 0.028 | 0.780 |
|  | 2 | -0.038 | 0.036 | 0.296 |  | 0.018 | 0.028 | 0.537 |
|  |  | *Attention Problems* | | | | | | |
| Interdaily Stability | 1 | 0.015 | 0.031 | 0.618 |  | 0.007 | 0.015 | 0.622 |
|  | 2 | 0.033 | 0.032 | 0.299 |  | 0.002 | 0.015 | 0.911 |
| Intradaily Variability | 1 | 0.161 | 0.033 | <0.001 |  | 0.023 | 0.018 | 0.189 |
|  | 2 | 0.165 | 0.035 | <0.001 |  | 0.024 | 0.018 | 0.188 |
| Onset least active 5 hours | 1 | -0.046 | 0.034 | 0.177 |  | -0.002 | 0.016 | 0.889 |
|  | 2 | -0.034 | 0.035 | 0.343 |  | -0.002 | 0.016 | 0.907 |
|  |  | *Rule-breaking Behavior* | | | | | | |
| Interdaily Stability | 1 | 0.028 | 0.031 | 0.376 |  | 0.015 | 0.034 | 0.662 |
|  | 2 | 0.037 | 0.032 | 0.250 |  | 0.003 | 0.035 | 0.936 |
| Intradaily Variability | 1 | 0.008 | 0.034 | 0.814 |  | 0.004 | 0.042 | 0.917 |
|  | 2 | 0.001 | 0.035 | 0.974 |  | -0.004 | 0.042 | 0.925 |
| Onset least active 5 hours | 1 | 0.014 | 0.035 | 0.685 |  | -0.016 | 0.038 | 0.661 |
|  | 2 | 0.015 | 0.036 | 0.671 |  | -0.012 | 0.038 | 0.745 |
|  |  | *Aggressive Behavior* | | | | | | |
| Interdaily Stability | 1 | 0.020 | 0.031 | 0.523 |  | 0.013 | 0.015 | 0.370 |
|  | 2 | 0.028 | 0.032 | 0.385 |  | 0.009 | 0.015 | 0.547 |
| Intradaily Variability | 1 | 0.024 | 0.034 | 0.481 |  | <0.001 | 0.018 | 0.987 |
|  | 2 | 0.019 | 0.035 | 0.584 |  | -0.002 | 0.018 | 0.925 |
| Onset least active 5 hours | 1 | -0.008 | 0.035 | 0.825 |  | -0.016 | 0.016 | 0.333 |
|  | 2 | -0.004 | 0.036 | 0.922 |  | -0.015 | 0.016 | 0.365 |
| Sleep characteristics and behavioral measurements were standardized to a mean of 0 and SD 1, ß coefficients represent change in SD. | | | | | | | | |
| Model 1 is corrected for age at actigraphy measurement, age at behavioral measurement and sex, model 2 is additionally corrected for gestational age at birth, child national origin, maternal education and maternal psychopathology | | | | | | | | |

| **Table S5. Exploratory analyses: associations of individual syndrome scales with reported sleep problems** | | | | | | | | |
| --- | --- | --- | --- | --- | --- | --- | --- | --- |
|  | model | ß | SE | p-value |  | ß | SE | p-value |
|  |  | 10-11 years | | |  | 13-14 years | | |
|  |  | *Social Problems* | | | | | | |
| Self-reported | 1 | 0.079 | 0.033 | 0.015 |  | 0.239 | 0.046 | <0.001 |
|  | 2 | 0.062 | 0.031 | 0.047 |  | 0.243 | 0.046 | <0.001 |
| Parent-reported | 1 | 0.214 | 0.032 | <0.001 |  | 0.178 | 0.050 | <0.001 |
|  | 2 | 0.166 | 0.032 | <0.001 |  | 0.169 | 0.050 | 0.001 |
|  |  | *Thought Problems* | | | | | | |
| Self-reported | 1 | 0.180 | 0.033 | <0.001 |  | 0.334 | 0.048 | <0.001 |
|  | 2 | 0.164 | 0.032 | <0.001 |  | 0.337 | 0.048 | <0.001 |
| Parent-reported | 1 | 0.313 | 0.032 | <0.001 |  | 0.353 | 0.051 | <0.001 |
|  | 2 | 0.272 | 0.032 | <0.001 |  | 0.348 | 0.052 | <0.001 |
| Sleep characteristics and behavioral measurements were standardized to a mean of 0 and SD 1, ß coefficients represent change in SD. | | | | | | | | |
| Model 1 is corrected for age at reported sleep measurement, age at behavioral measurement and sex, model 2 is additionally corrected for gestational age at birth, child national origin, maternal education and maternal psychopathology | | | | | | | | |

| **Table S6. Exploratory analyses: associations of syndrome scales with actigraphic sleep patterns** | | | | | | | | |
| --- | --- | --- | --- | --- | --- | --- | --- | --- |
|  | model | ß | SE | p-value |  | ß | SE | p-value |
|  |  | 10-11 years | | |  | 13-14 years | | |
|  |  | *Social Problems* | | | | | | |
| Sleep duration | 1 | 0.055 | 0.032 | 0.085 |  | 0.031 | 0.027 | 0.251 |
|  | 2 | 0.088 | 0.033 | 0.009 |  | 0.036 | 0.027 | 0.187 |
| Sleep onset | 1 | -0.078 | 0.029 | 0.007 |  | -0.031 | 0.022 | 0.173 |
|  | 2 | -0.111 | 0.030 | <0.001 |  | -0.033 | 0.023 | 0.152 |
| Sleep onset latency | 1 | -0.025 | 0.032 | 0.441 |  | -0.010 | 0.013 | 0.420 |
|  | 2 | -0.030 | 0.034 | 0.380 |  | -0.016 | 0.013 | 0.205 |
| Sleep efficiency | 1 | 0.051 | 0.031 | 0.097 |  | -0.003 | 0.028 | 0.914 |
|  | 2 | 0.056 | 0.033 | 0.085 |  | 0.005 | 0.028 | 0.858 |
| Nocturnal waking times | 1 | -0.070 | 0.038 | 0.066 |  | 0.022 | 0.030 | 0.472 |
|  | 2 | -0.059 | 0.040 | 0.138 |  | 0.025 | 0.031 | 0.418 |
|  |  | *Thought Problems* | | | | | | |
| Sleep duration | 1 | -0.047 | 0.031 | 0.133 |  | 0.011 | 0.023 | 0.629 |
|  | 2 | -0.038 | 0.033 | 0.248 |  | 0.012 | 0.023 | 0.608 |
| Sleep onset | 1 | 0.014 | 0.029 | 0.612 |  | -0.019 | 0.019 | 0.321 |
|  | 2 | <0.001 | 0.030 | 0.989 |  | -0.017 | 0.019 | 0.387 |
| Sleep onset latency | 1 | -0.009 | 0.032 | 0.773 |  | -0.002 | 0.011 | 0.870 |
|  | 2 | -0.008 | 0.033 | 0.804 |  | -0.005 | 0.011 | 0.620 |
| Sleep efficiency | 1 | 0.040 | 0.030 | 0.188 |  | -0.039 | 0.024 | 0.097 |
|  | 2 | 0.034 | 0.032 | 0.283 |  | -0.036 | 0.024 | 0.136 |
| Nocturnal waking times | 1 | -0.067 | 0.037 | 0.070 |  | 0.036 | 0.026 | 0.166 |
|  | 2 | -0.054 | 0.039 | 0.166 |  | 0.035 | 0.026 | 0.178 |
| Sleep characteristics and behavioral measurements were standardized to a mean of 0 and SD 1, ß coefficients represent change in SD. | | | | | | | | |
| Model 1 is corrected for age at actigraphy measurement, age at behavioral measurement and sex, model 2 is additionally corrected for gestational age at birth, child national origin, maternal education and maternal psychopathology | | | | | | | | |

| **Table S7. Exploratory analyses: associations of syndrome scales and the 24h activity rhythm, measured by actigraphy** | | | | | | | | |
| --- | --- | --- | --- | --- | --- | --- | --- | --- |
|  | model | ß | SE | p-value |  | ß | SE | p-value |
|  |  | 10-11 years | | |  | 13-14 years | | |
|  |  | *Social Problems* | | | | | | |
| Interdaily Stability | 1 | 0.014 | 0.032 | 0.664 |  | 0.035 | 0.027 | 0.204 |
|  | 2 | 0.028 | 0.034 | 0.409 |  | 0.020 | 0.028 | 0.479 |
| Intradaily Variability | 1 | 0.117 | 0.035 | 0.001 |  | 0.060 | 0.033 | 0.073 |
|  | 2 | 0.120 | 0.037 | 0.001 |  | 0.055 | 0.034 | 0.104 |
| Onset least active 5 hours | 1 | -0.028 | 0.036 | 0.435 |  | -0.036 | 0.030 | 0.229 |
|  | 2 | -0.023 | 0.038 | 0.533 |  | -0.030 | 0.030 | 0.320 |
|  |  | *Thought Problems* | | | | | | |
| Interdaily Stability | 1 | 0.021 | 0.031 | 0.504 |  | 0.033 | 0.023 | 0.164 |
|  | 2 | 0.031 | 0.033 | 0.343 |  | 0.024 | 0.023 | 0.297 |
| Intradaily Variability | 1 | 0.030 | 0.034 | 0.387 |  | 0.036 | 0.028 | 0.203 |
|  | 2 | 0.031 | 0.036 | 0.387 |  | 0.031 | 0.029 | 0.277 |
| Onset least active 5 hours | 1 | -0.064 | 0.035 | 0.068 |  | 0.003 | 0.026 | 0.918 |
|  | 2 | -0.053 | 0.036 | 0.150 |  | 0.010 | 0.026 | 0.708 |
| Sleep characteristics and behavioral measurements were standardized to a mean of 0 and SD 1, ß coefficients represent change in SD. | | | | | | | | |
| Model 1 is corrected for age at actigraphy measurement, age at behavioral measurement and sex, model 2 is additionally corrected for gestational age at birth, child national origin, maternal education and maternal psychopathology | | | | | | | | |

| **Table S8. Associations of mental health problems and nocturnal waking times as count variable** | | | | | | | | |
| --- | --- | --- | --- | --- | --- | --- | --- | --- |
| Sleep patterns | model | ß | SE | p-value |  | ß | SE | p-value |
|  |  | 10-11 years | | |  | 13-14 years | | |
|  |  | *Internalizing problems* | | | | | | |
| Nocturnal waking times | 1 | -0.002 | 0.023 | 0.929 |  | 0.058 | 0.034 | 0.092 |
|  | 2 | 0.010 | 0.024 | 0.688 |  | 0.060 | 0.035 | 0.089 |
|  |  | *Externalizing problems* | | | | | | |
| Nocturnal waking times | 1 | -0.010 | 0.024 | 0.674 |  | -0.010 | 0.042 | 0.821 |
|  | 2 | -0.003 | 0.025 | 0.891 |  | -0.003 | 0.043 | 0.943 |
|  |  | *Dysregulation profile* | | | | | | |
| Nocturnal waking times | 1 | -0.014 | 0.024 | 0.575 |  | 0.030 | 0.036 | 0.411 |
|  | 2 | -0.005 | 0.026 | 0.858 |  | 0.033 | 0.037 | 0.369 |
| Model 1 is corrected for age at actigraphy measurement, age at behavioral measurement and sex, model 2 is additionally corrected for gestational age at birth, child national origin, maternal education and maternal psychopathology | | | | | | | | |

*Additional Figures*

*
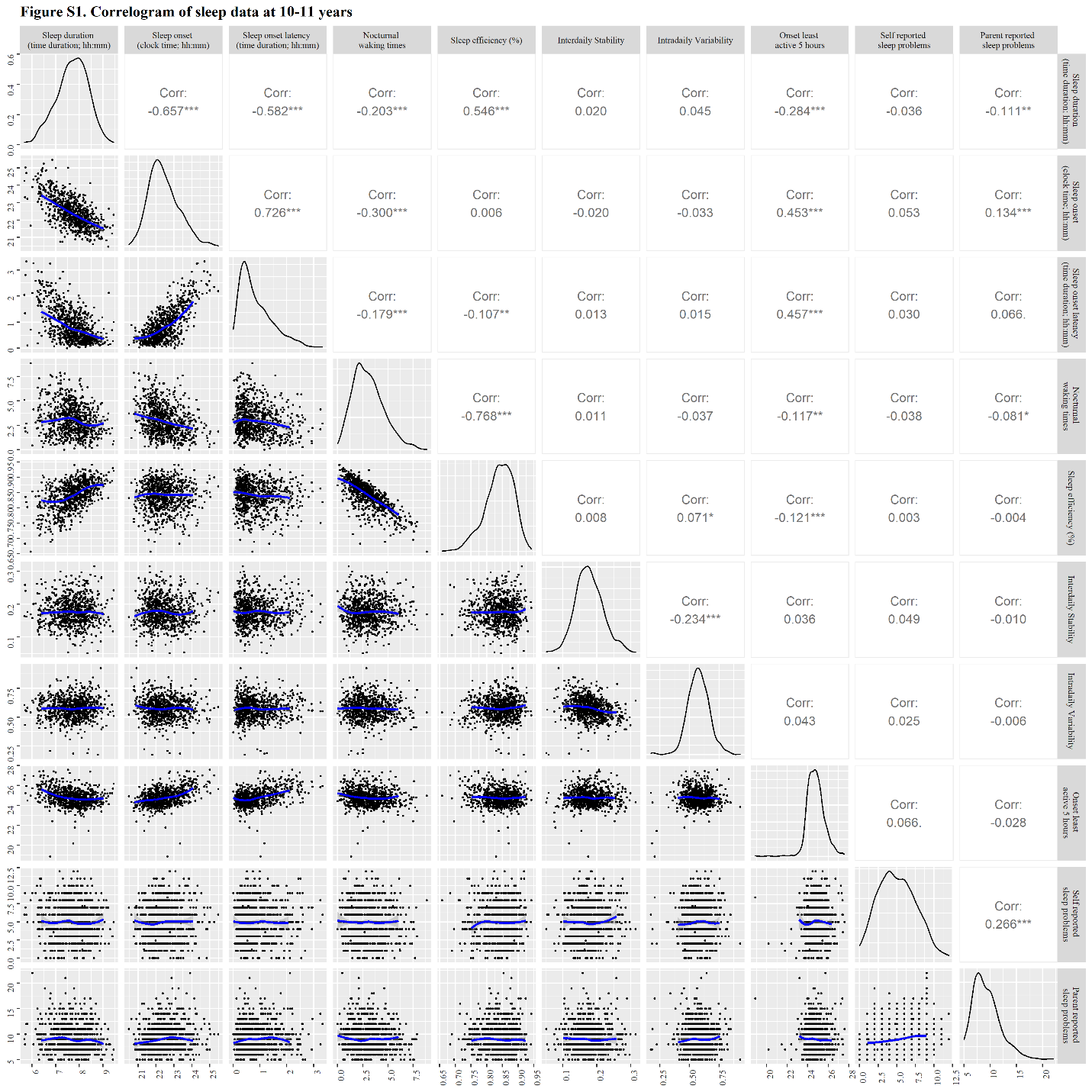
*

**
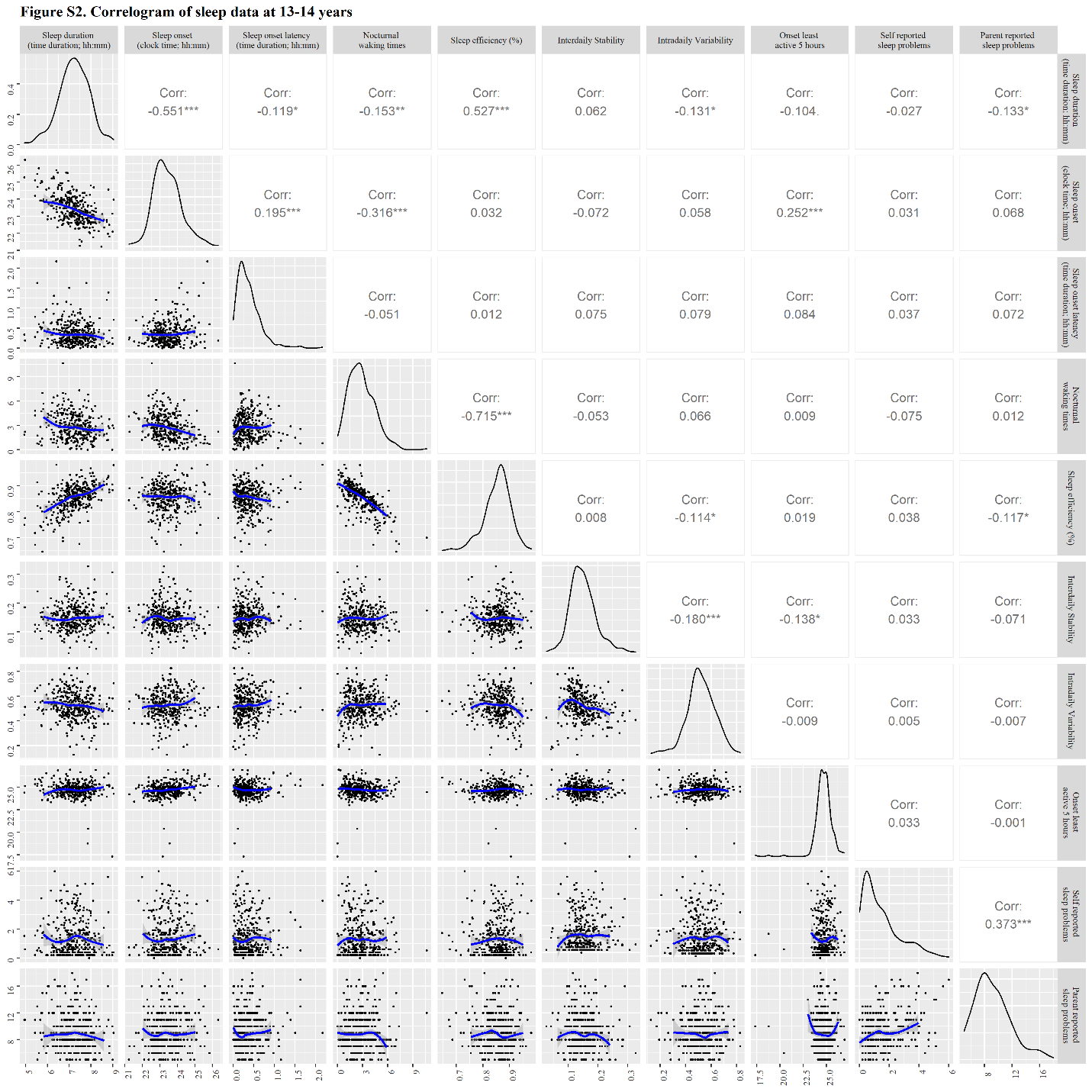
**

**
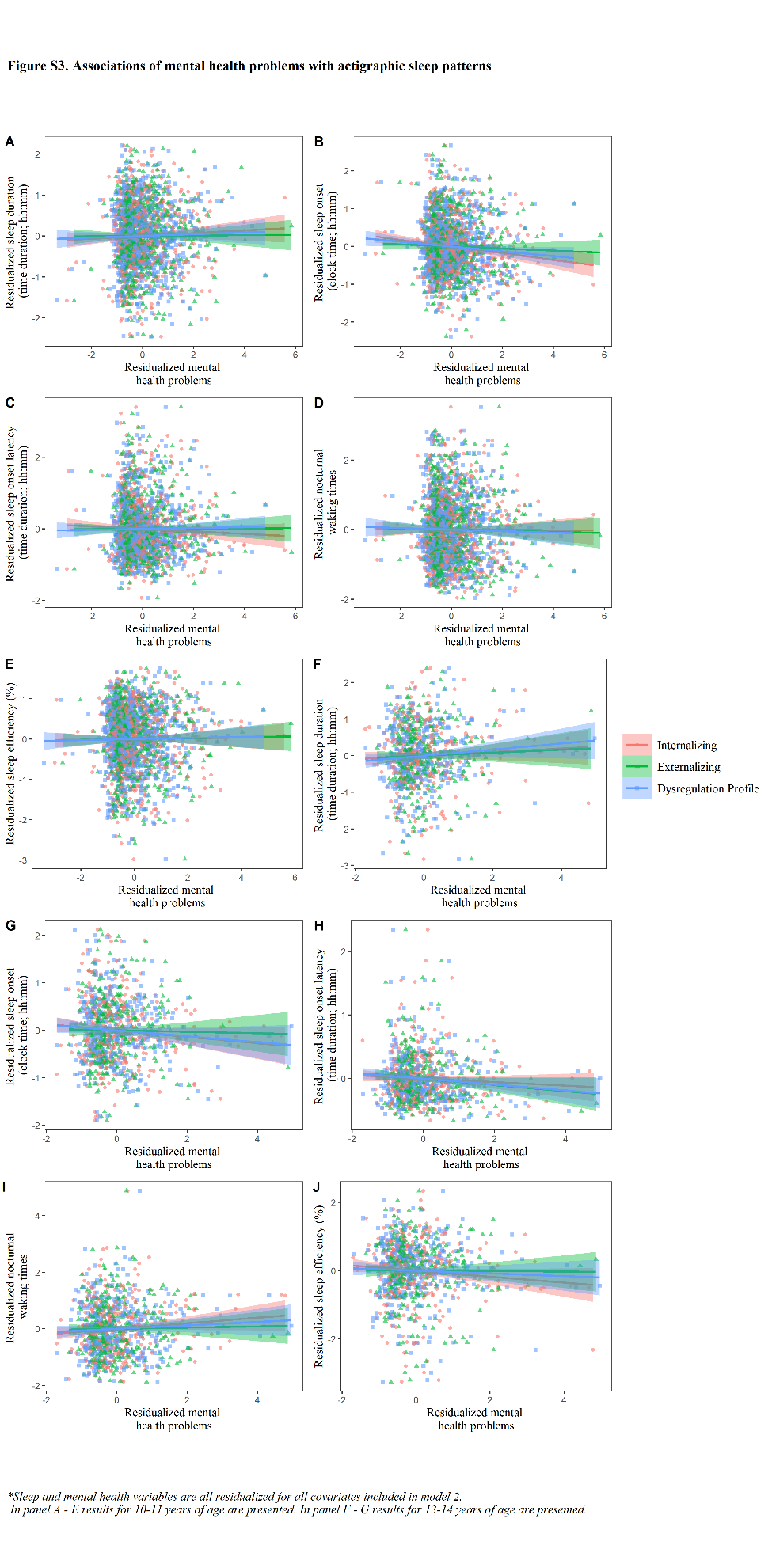
**

**
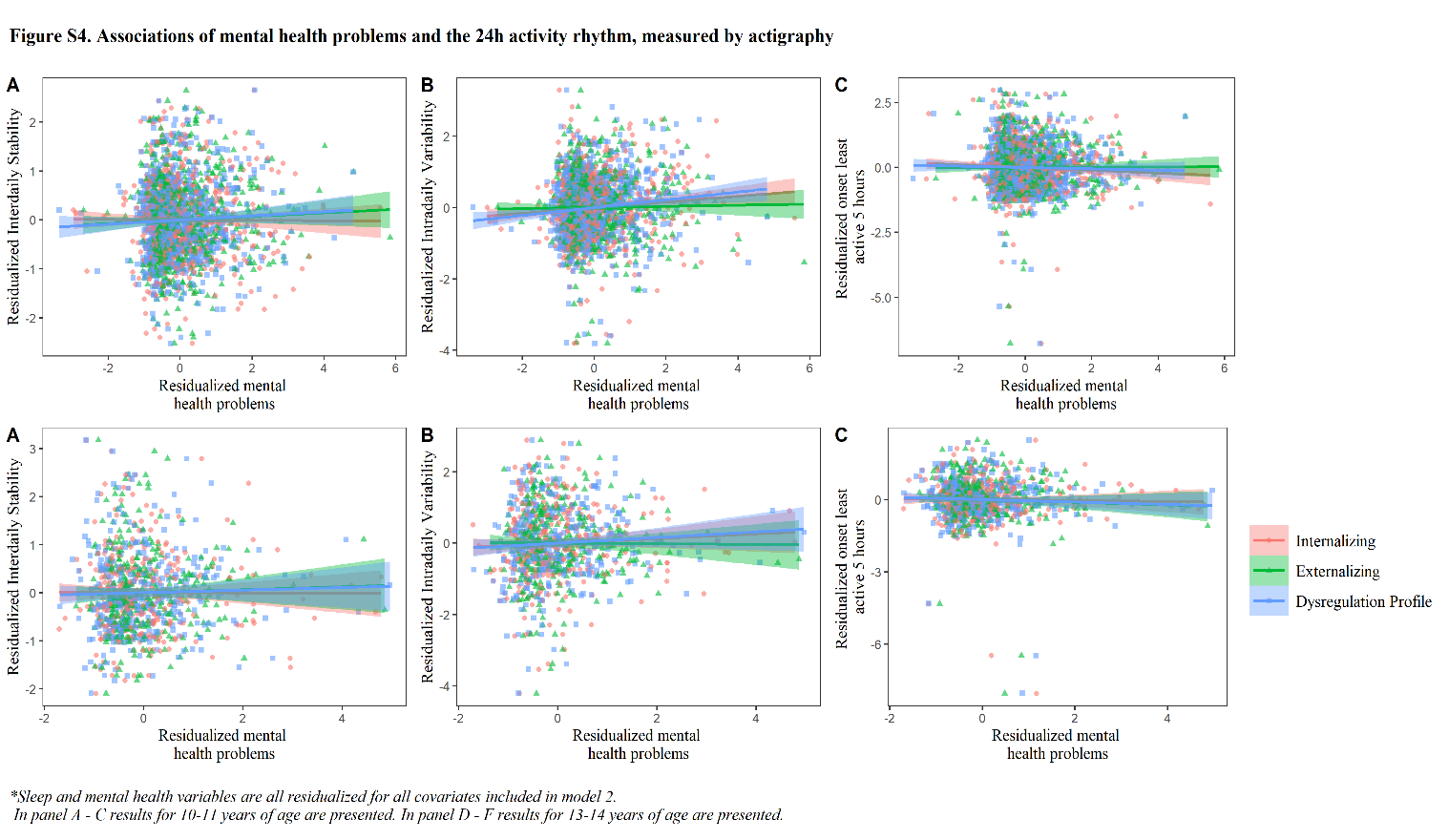
**
